# Supplementary material for: Hierarchies and Dominance Behaviors in European Pond Turtle (Emys orbicularis galloitalica) Hatchlings in a Controlled Environment
Source: Animals (Basel). 2020 Aug 26;10(9):1510. doi: 10.3390/ani10091510 (PMC7552232; doi:10.3390/ani10091510)
Supplement: Supplementary file 1 [file animals-10-01510-s001.pdf]

animals

Supplementary materials: Hierarchies and dominance behaviors in European Pond Turtle (*Emys orbicularis galloitalica*) hatchlings in a controlled environment

Simone Masin [1], Luciano Bani [1,*], Davide Vardanega [1], Norberto Chiodini [2] and Valerio Orioli [1]

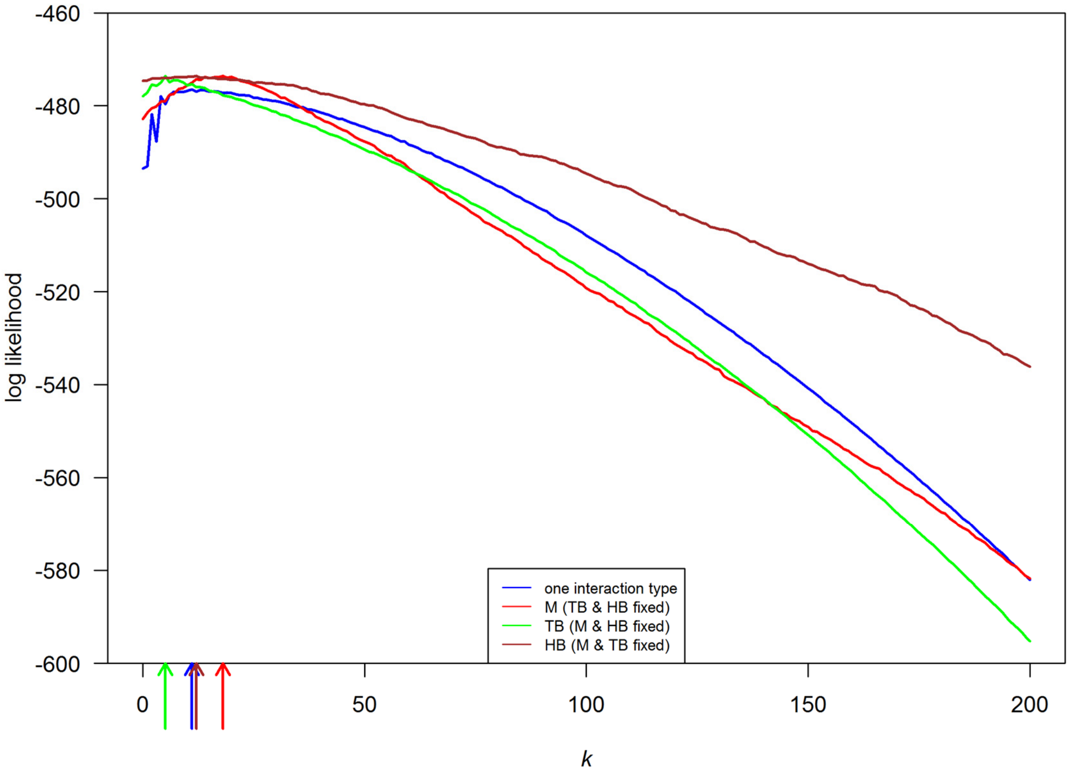

Figure S1. Comparison between constant k and interaction-dependent k models for group one.

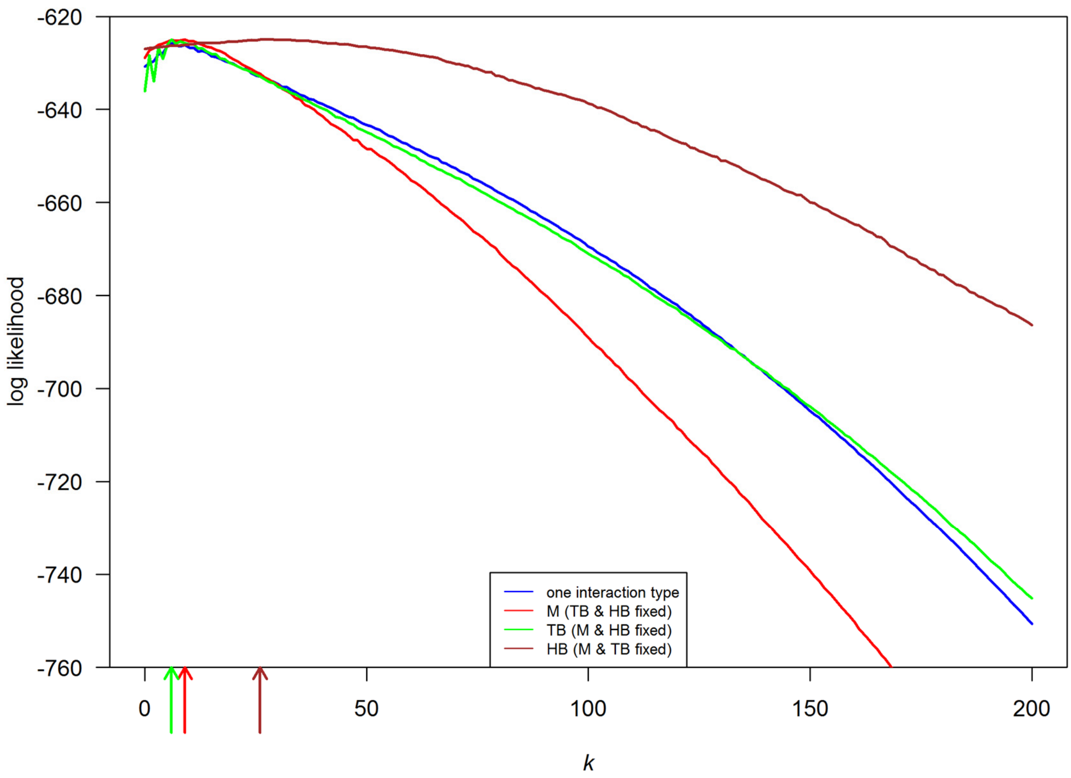

Figure S2. Comparison between constant *k* and interaction-dependent *k* models for group two.

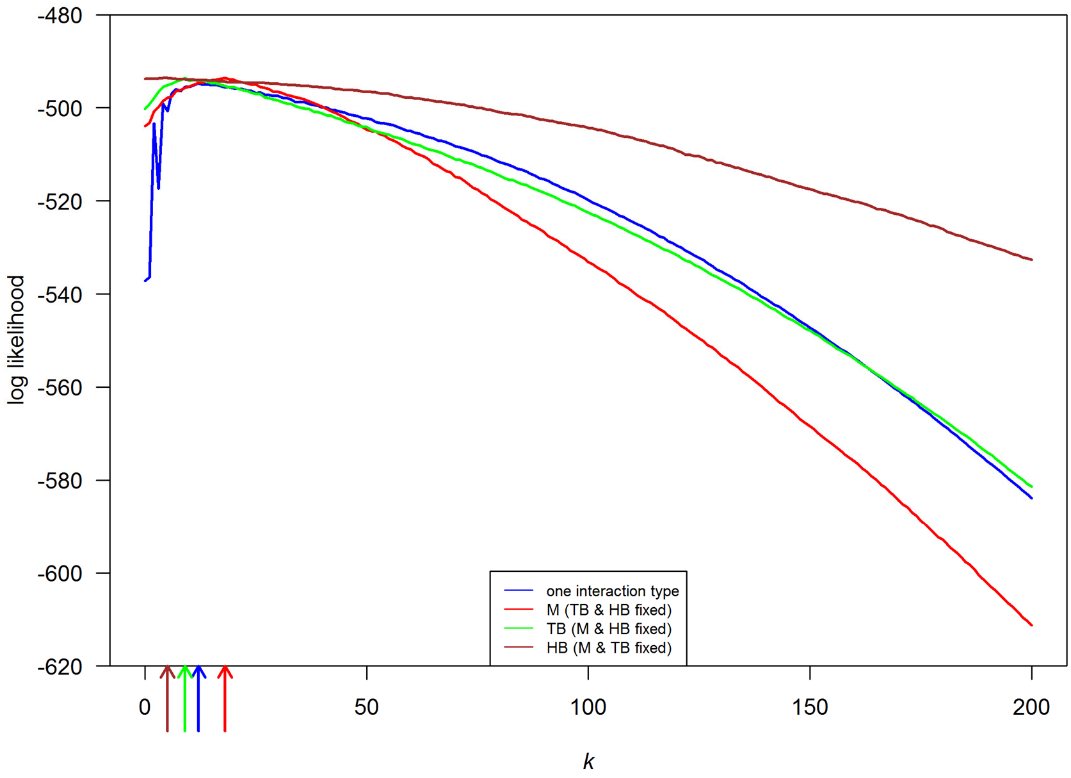

Figure S3. Comparison between constant *k* and interaction-dependent *k* models for group three.

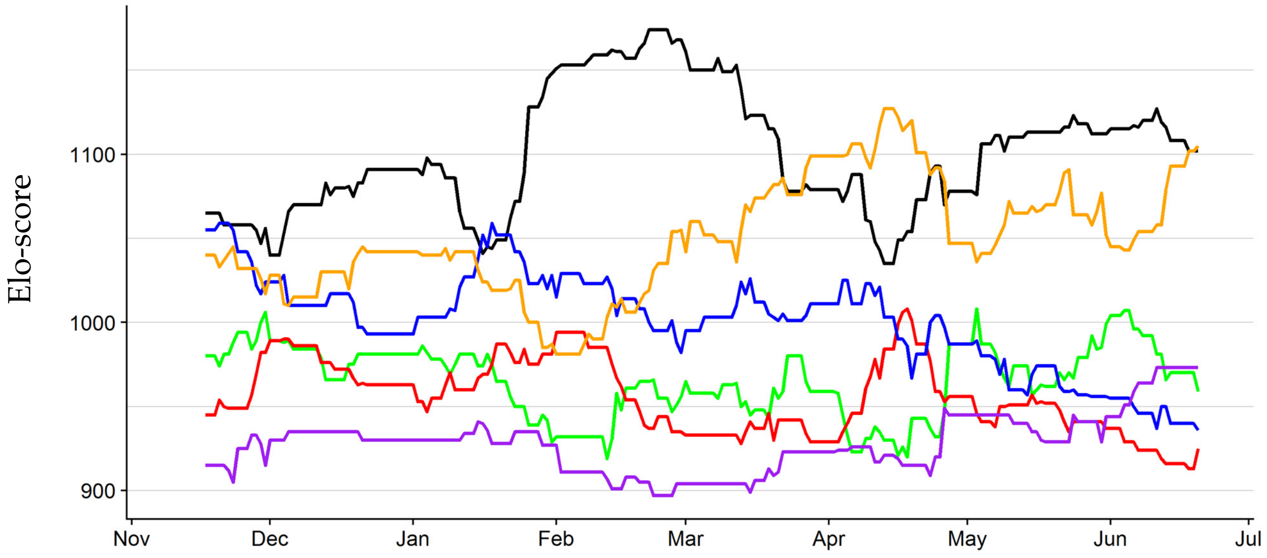

Figure S4. Elo-scores dynamics with optimized starting values for group one. For color-individual association see Figure 2 in the main text.

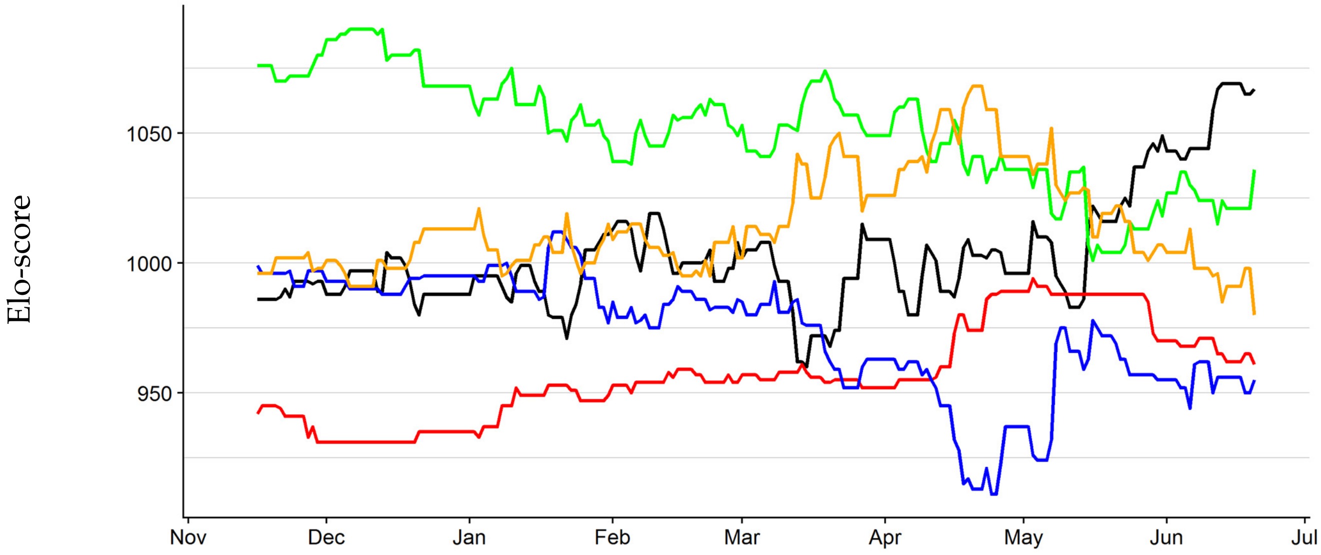

Figure S5. Elo-scores dynamics with optimized starting values for group two. For color-individual association see Figure 2 in the main text.

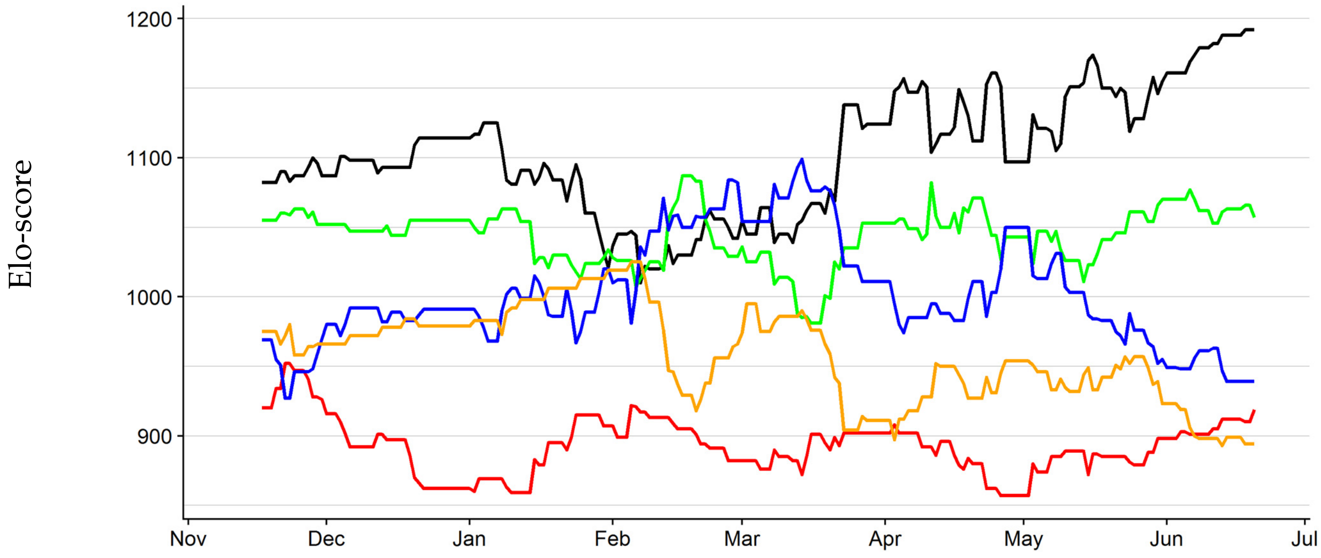

Figure S6. Elo-scores dynamics with optimized starting values for group three. For color-individual association see Figure 2 in the main text.

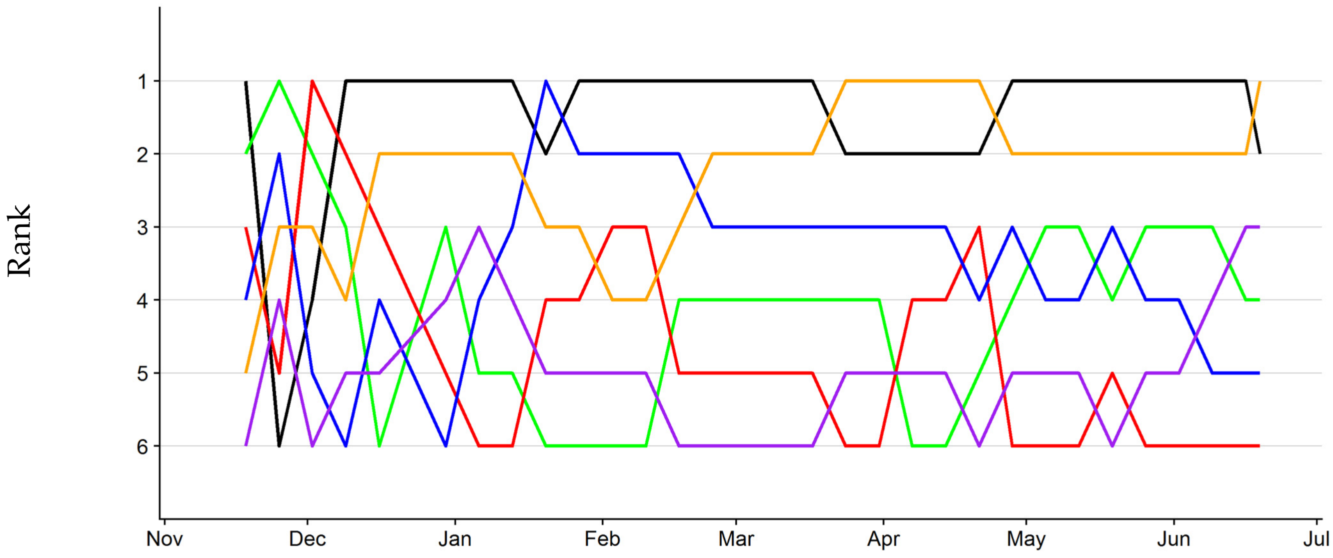

Figure S7. Weekly rank dynamics for group one. Ranks corresponded to the ranked Elo-scores at Table 2. in the main text.

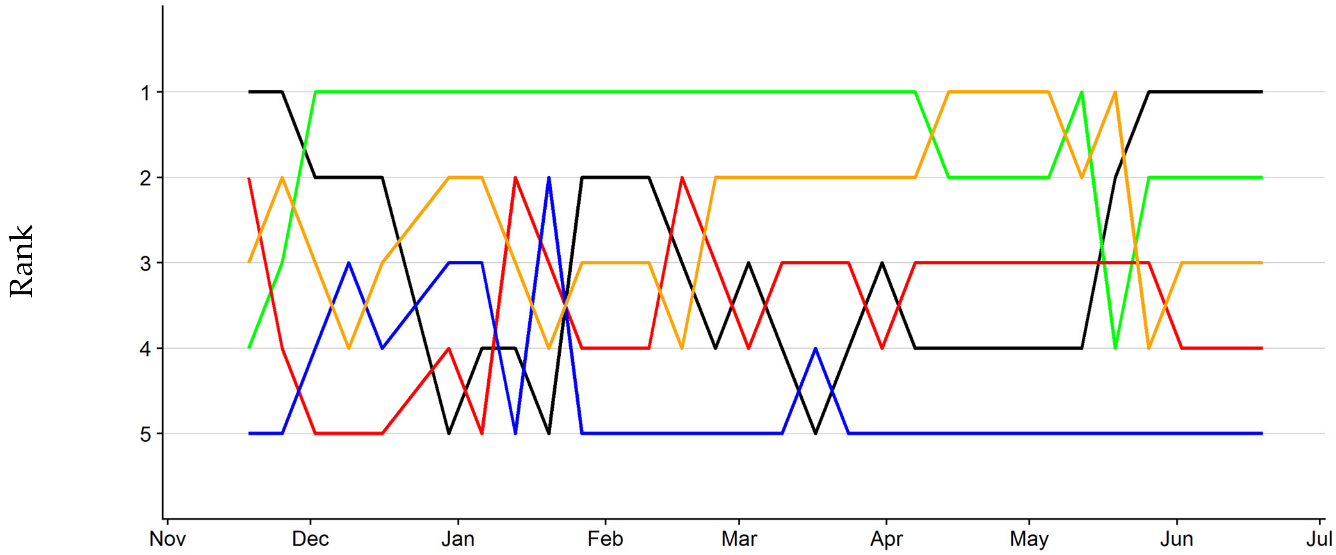

Figure S8. Weekly rank dynamics for group two. Ranks corresponded to the ranked Elo-scores at the end of each week. For color-individual association see Figure 2 in the main text.

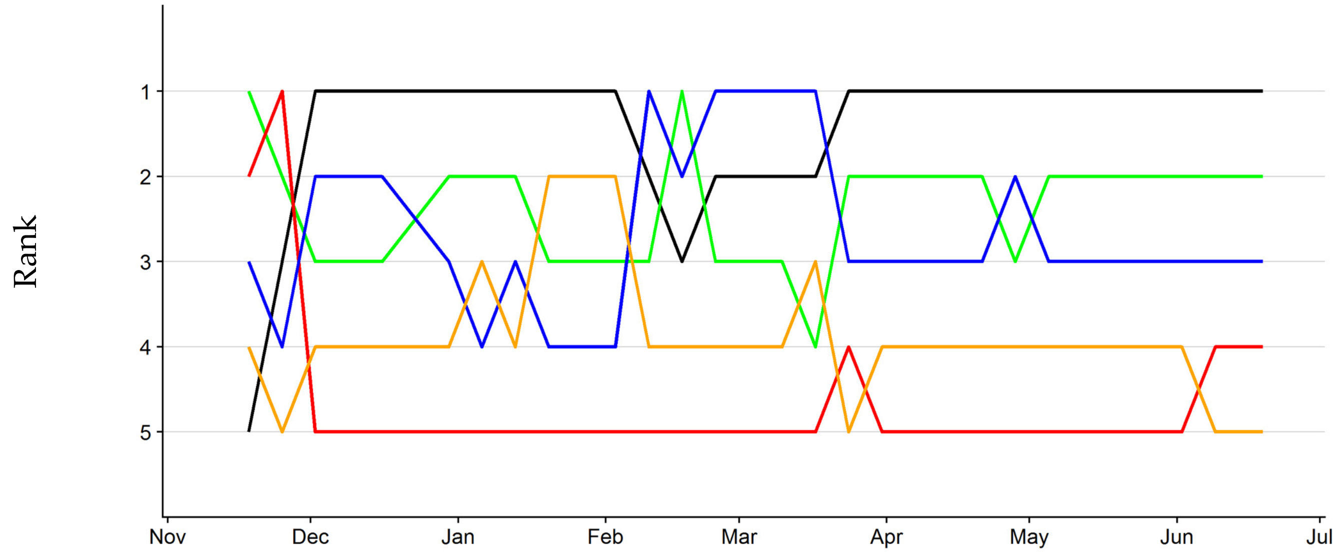

Figure S9. Weekly rank dynamics for group three. Ranks corresponded to the ranked Elo-scores at the end of each week. For color-individual association see Figure 2 in the main text.

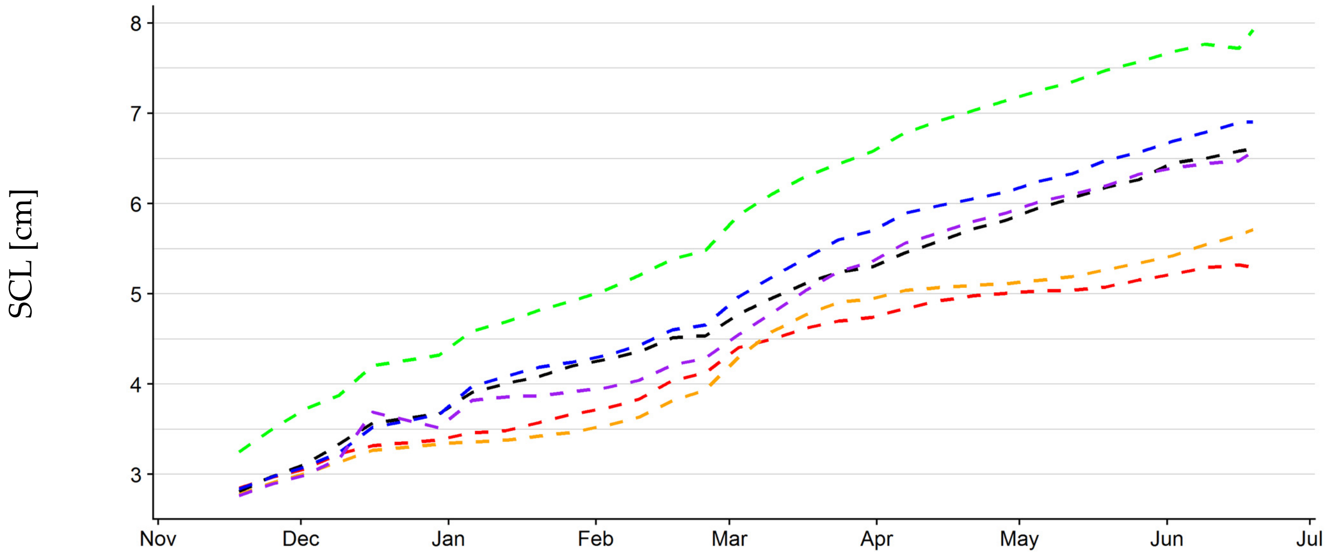

Figure S10. Straight Carapace Length increase for individuals owing to group one. For color-individual association see Figure 2 in the main text.

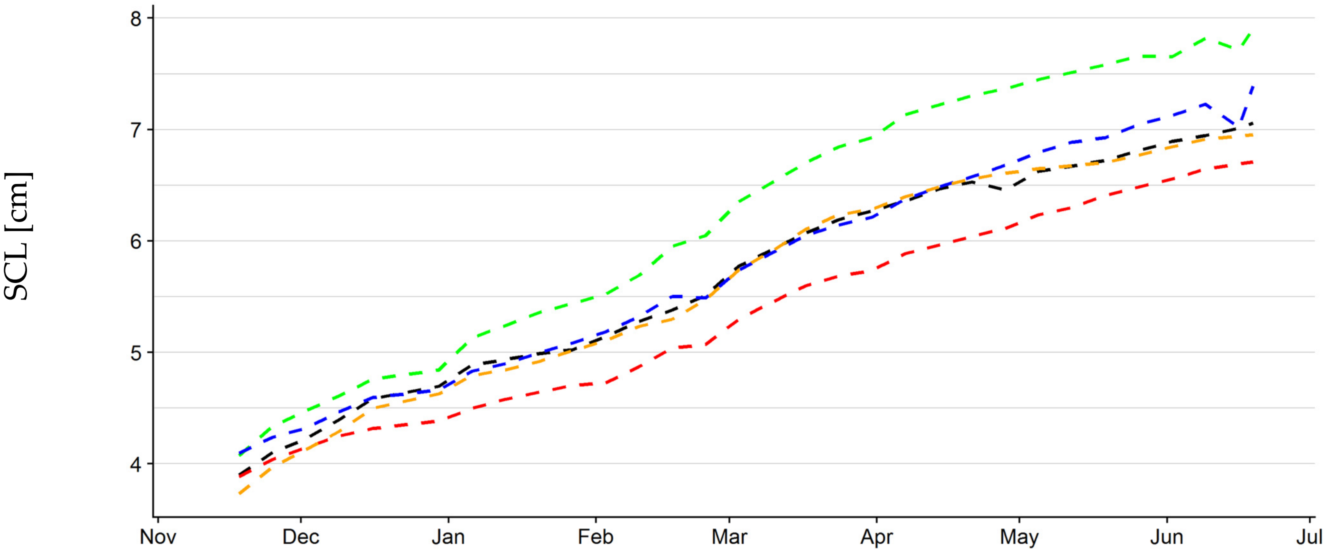

Figure S11. Straight Carapace Length increase for individuals owing to group two. For color-individual association see Figure 2 in the main text.

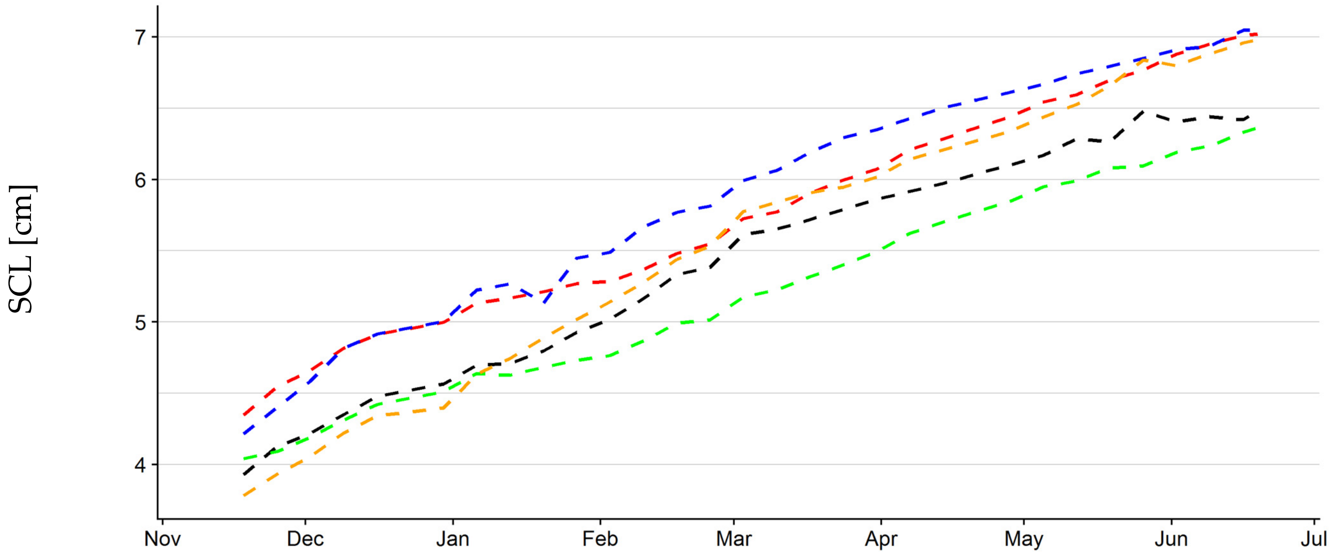

Figure S12. Straight Carapace Length increase for individuals owing to group three. For color-individual association see Figure 2 in the main text.

Table S1. Linear mixed models of the difference in Elo-score on the difference in SCL from the higher ranked and the larger individual, respectively, at the end of each week. The identity of individuals was used as random effect for both intercept and β; SD: Standard deviation of random effect.

Group	Intercept			SCL Difference			SD of Random Effect	
	Value	SE	*p*-Value	β	SE	*p*-Value	Intercept	SCL difference
1	−5.21	36.85	0.893	63.49	61.66	0.355	85.92	148.9
2	23.67	6.37	0.014	58.62	32.92	0.168	11.52	72.58
3	110.7	46.11	0.078	-61.15	93.16	0.543	96.57	186.7
